# Supplementary material for: Mpox: Clinical Outcomes and Impact of Vaccination in People with and without HIV: A Population-Wide Study
Source: Microorganisms. 2023 Nov 3;11(11):2701. doi: 10.3390/microorganisms11112701 (PMC10673392; doi:10.3390/microorganisms11112701)
Supplement: Supplementary file 1 [file microorganisms-11-02701-s001.zip › microorganisms-2614898-supplementary.pdf]

Supplementary material

Table S1. Description of ICD-10 codes used to define the comorbidities assess in the study (based on the Swedish National Study of Aging and Care in Kungsholmen (SNAC-K) coding).

**ISCHEMIC HEART DISEASE**	
**Included ICD-10 codes and labels**	
I20	Angina pectoris
I21	Acute myocardial infarction
I22	Subsequent myocardial infarction
I24	Other acute ischaemic heart diseases
I25	Chronic ischaemic heart disease
Z951	Presence of aortocoronary bypass graft
Z955	Presence of coronary angioplasty implant and graft
Additional parameters	Use of organic nitrates (C01DA) or ranolazine (C01EB18)
**HEART FAILURE**	
**Included ICD-10 codes and labels**	
I110	Hypertensive heart disease with (congestive) heart failure
I130	Hypertensive heart and renal disease with (congestive) heart failure
I132	Hypertensive heart and renal disease with both (congestive) heart failure and renal failure
I27	Other pulmonary heart diseases
I280	Arteriovenous fistula of pulmonary vessels
I42	Cardiomyopathy
I43	Cardiomyopathy in diseases classified elsewhere
I50	Heart failure
I515	Myocardial degeneration
I517	Cardiomegaly
I528	Other heart disorders in other diseases classified elsewhere
Z941	Heart transplant status
Z943	Heart and lungs transplant status
**CEREBROVASCULAR DISEASE**	
**Included ICD-10 codes and labels**	
G45	Transient cerebral ischaemic attacks and related syndromes
G46	Vascular syndromes of brain in cerebrovascular diseases
I60	Subarachnoid haemorrhage
I61	Intracerebral haemorrhage
I62	Other nontraumatic intracranial haemorrhage
I63	Cerebral infarction
I64	Stroke, not specified as haemorrhage or infarction
I67	Other cerebrovascular diseases
I69	Sequelae of cerebrovascular disease
**PERIPHERAL VASCULAR DISEASE**	
**Included ICD-10 codes and labels**	
I702	Atherosclerosis of arteries of extremities
I73	Other peripheral vascular diseases
I792	Peripheral angiopathy in diseases classified elsewhere
I798	Other disorders of arteries, arterioles and capillaries in diseases classified elsewhere
Additional parameters	Use of cilostazol (B01AC23)
**Excluded ICD-10 codes and labels**	
I731	Thromboangiitis obliterans [Buerger]
I738	Other specified peripheral vascular diseases
**CHRONIC KIDNEY DISEASES**	
**Included ICD-10 codes and labels**	
I120	Hypertensive renal disease with renal failure

I130	Hypertensive heart and renal disease with (congestive) heart failure
I131	Hypertensive heart and renal disease with renal failure
I132	Hypertensive heart and renal disease with both (congestive) heart failure and renal failure
I139	Hypertensive heart and renal disease, unspecified
N01	Rapidly progressive nephritic syndrome
N03	Chronic nephritic syndrome
N04	Nephrotic syndrome
N05	Unspecified nephritic syndrome
N07	Hereditary nephropathy, not elsewhere classified
N08	Glomerular disorders in diseases classified elsewhere
N11	Chronic tubulo-interstitial nephritis
N183	Chronic kidney disease, stage 3
N184	Chronic kidney disease, stage 4
N185	Chronic kidney disease, stage 5
N189	Chronic kidney disease, unspecified
Q60	Renal agenesis and other reduction defects of kidney
Q611	Polycystic kidney, autosomal recessive
Q612	Polycystic kidney, autosomal dominant
Q613	Polycystic kidney, unspecified
Q614	Renal dysplasia
Q615	Medullary cystic kidney
Q618	Other cystic kidney diseases
Q619	Cystic kidney disease, unspecified
Z905	Acquired absence of kidney
Z940	Kidney transplant status
Additional parameters	Glomerular filtration rate <60 ml/min/1.73m2 (assessed using the CKD-EPI equation)[2]
**CHRONIC LIVER DISEASES**	
**Included ICD-10 codes and labels**	
B18	Chronic viral hepatitis
K70	Alcoholic liver disease
K713	Toxic liver disease with chronic persistent hepatitis
K714	Toxic liver disease with chronic lobular hepatitis
K715	Toxic liver disease with chronic active hepatitis
K717	Toxic liver disease with fibrosis and cirrhosis of liver
K721	Chronic hepatic failure
K73	Chronic hepatitis, not elsewhere classified
K74	Fibrosis and cirrhosis of liver
K753	Granulomatous hepatitis, not elsewhere classified
K754	Autoimmune hepatitis
K758	Other specified inflammatory liver diseases
K761	Chronic passive congestion of liver
K766	Portal hypertension
K767	Hepatorenal syndrome
K778	Liver disorders in other diseases classified elsewhere
Q446	Cystic disease of liver
Z944	Liver transplant status
**Excluded ICD-10 codes and labels**	
K700	Alcoholic fatty liver
K701	Alcoholic hepatitis
**COPD, EMPHYSEMA, CHRONIC BRONCHITIS**	
**Included ICD-10 codes and labels**	
J41	Simple and mucopurulent chronic bronchitis
J42	Unspecified chronic bronchitis
J43	Emphysema
J44	Other chronic obstructive pulmonary disease
J47	Bronchiectasis

Additional parameters	Use of anticholinergics (R03BB)
**DEMENTIA**	
**Included ICD-10 codes and labels**	
F00	Dementia in Alzheimer disease
F01	Vascular dementia
F02	Dementia in other diseases classified elsewhere
F03	Unspecified dementia
F051	Delirium superimposed on dementia
G30	Alzheimer disease
G31	Other degenerative diseases of nervous system, not elsewhere classified
Additional parameters	Diagnostic and Statistical Manual of Mental Disorders, Third Edition, Revised (assessed by two different physicians, and a third one in case of disagreement)<br>Use of anticholinesterases (N06DA) or memantine (N06DX01)
**DIABETES**	
**Included ICD-10 codes and labels**	
E10	Insulin-dependent diabetes mellitus
E11	Non-insulin-dependent diabetes mellitus
E13	Other specified diabetes mellitus
E14	Unspecified diabetes mellitus
E891	Postprocedural hypoinsulinaemia
Additional parameters	Glycated hemoglobin (A1C) ≥6.5%<br>Use of antidiabetics (A10)
**HEMATOLOGICAL NEOPLASMS**	
**Included ICD-10 codes and labels**	
C81	Hodgkin lymphoma
C82	Follicular lymphoma
C83	Non-follicular lymphoma
C84	Mature T/NK-cell lymphomas
C85	Other and unspecified types of non-Hodgkin lymphoma
C86	Other specified types of T/NK-cell lymphoma
C88	Malignant immunoproliferative diseases
C90	Multiple myeloma and malignant plasma cell neoplasms
C91	Lymphoid leukaemia
C92	Myeloid leukaemia
C93	Monocytic leukaemia
C94	Other leukaemias of specified cell type
C95	Leukaemia of unspecified cell type
C96	Other and unspecified malignant neoplasms of lymphoid, haematopoietic and related tissue
**SOLID NEOPLASMS**	
**Included ICD-10 codes and labels**	
C	Malignant neoplasms
D00	Carcinoma in situ of oral cavity, oesophagus and stomach
D01	Carcinoma in situ of other and unspecified digestive organs
D02	Carcinoma in situ of middle ear and respiratory system
D03	Melanoma in situ
D04	Carcinoma in situ of skin
D05	Carcinoma in situ of breast
D06	Carcinoma in situ of cervix uteri
D07	Carcinoma in situ of other and unspecified genital organs
D09	Carcinoma in situ of other and unspecified sites
D320	Benign neoplasm: Cerebral meninges
D321	Benign neoplasm: Spinal meninges
D329	Benign neoplasm: Meninges, unspecified
D330	Benign neoplasm: Brain, supratentorial
D331	Benign neoplasm: Brain, infratentorial
D332	Benign neoplasm: Brain, unspecified
D333	Benign neoplasm: Cranial nerves

D334	Benign neoplasm: Spinal cord
Q85	Phakomatoses, not elsewhere classified
**Excluded ICD-10 codes and labels**	
C81	Hodgkin lymphoma
C82	Follicular lymphoma
C83	Non-follicular lymphoma
C84	Mature T/NK-cell lymphomas
C85	Other and unspecified types of non-Hodgkin lymphoma
C86	Other specified types of T/NK-cell lymphoma
C88	Malignant immunoproliferative diseases
C90	Multiple myeloma and malignant plasma cell neoplasms
C91	Lymphoid leukaemia
C92	Myeloid leukaemia
C93	Monocytic leukaemia
C94	Other leukaemias of specified cell type
C95	Leukaemia of unspecified cell type
C96	Other and unspecified malignant neoplasms of lymphoid, haematopoietic and related tissue

Table S2. Additional clinical and drug-related parameters used in SNAC-K to define specific chronic conditions.

Condition	Clinical and drug-related parameters
**Chronic kidney diseases**	Glomerular filtration rate <60 ml/min/1.73m2 (assessed using the CKD-EPI equation)[1]
**COPD, Emphysema, Chronic Bronchitis**	Use of anticholinergics (R03BB)
**Dementia**	Diagnostic and Statistical Manual of Mental Disorders, Third Edition, Revised[2] (assessed by two different physicians, and a third one in case of disagreement)<br>Use of anticholinesterases (N06DA) or memantine (N06DX01)
**Diabetes**	Glycated hemoglobin (A1C) ≥6.5%[3]<br>Use of antidiabetics (A10)
**Ischemic heart disease**	Use of organic nitrates (C01DA) or ranolazine (C01EB18)
**Peripheral vascular disease**	Use of cilostazol (B01AC23)

[*]The ATC codes corresponding to each drug are shown in brackets. Only those drugs that can be unequivocally linked to chronic conditions were considered. The selection of ATC codes was based on a literature review and the clinical judgment of physicians.
NOTE: The criteria presented in this table were used in addition to the diagnoses assigned in SNAC-K.

[1] Levey AS, Stevens LA, Schmid CH, Zhang YL, Castro AF, Feldman HI, et al. A new equation to estimate glomerular filtration rate. Ann Intern Med. 2009;150(9):604–12.
[2] American Psychiatric Association. Diagnostic and statistical manual of mental disorders (3rd ed., text rev.). Washington, DC; 1987.
[3] American Diabetes Association. Standards of Medical Care in Diabetes. Diabetes Care. 2016;39(Suppl 1):S4-5.
[4] European Association for Cardiovascular Prevention & Rehabilitation, Reiner Z, Catapano AL, De Backer G, Graham I, Taskinen MR, et al. ESC/EAS Guidelines for the management of dyslipidaemias: the Task Force for the management of dyslipidaemias of the European Society of Cardiology (ESC) and the European Atherosclerosis Society (EAS). Eur Heart J. 2011;32(14):1769–818.
[5] Mancia G, De Backer G, Dominiczak A, Cifkova R, Fagard R, Germano G, et al. 2007 Guidelines for the management of arterial hypertension: The Task Force for the Management of Arterial Hypertension of the European Society of Hypertension (ESH) and of the European Society of Cardiology (ESC). Eur Heart J. 2007;28(12):1462–536.

Table S3. Sexually transmitted infections in people with mpox (with and without HIV) and in people with HIV and without mpox

Sexually transmitted infections (STIs)	People with mpox N=2122				PWH without mpox[2] N=22477	
	All mpox cases	PWoH (n=1280)	PWH¨ (n=842)	p-value		p-value
Total number of STIs at the moment of mpox, n (%)				0.207		
1	305 (14.4)	196 (15.3)	109 (13.0)		NA	
2	43 (2.0)	21 (1.6)	22 (2.6)		NA	
>2	2 (0.09)	1 (0.08)	1 (0.1)		NA	
Total number of STIs 2 years before mpox, n (%)				0.129		
1	448 (21.1)	265 (20.7)	183 (21.7)		1319 (5.9)	<0.0001
2	246 (11.6)	140 (11.0)	106 (12.6)		508 (2.3)	<0.0001
3	124 (5.9)	76 (5.9)	48 (5.7)		172 (0.8)	<0.0001
>3	100 (4.7)	60 (4.7)	40 (4.8)		102 (	<0.0001
Syphilis						
Syphilis at mpox diagnosis, n (%)[1]	34 (1.6)	15 (1.2)	19 (2.3)	0.052	NA	
Syphilis in the 2 years before mpox, n (%)	205 (9.7)	96 (7.5)	109 (13.0)	<0.0001	697 (3.1)	<0.0001
Number of syphilis events in the 2 years before mpox, n (%)						
1	182 (8.6)	89 (7.0)	93 (11.1)		577 (2.6)	
2	20 (0.9)	6 (0.5)	14 (1.7)		110 (0.5)	
>2	3 (0.1)	1 (0.08)	2 (0.24)		10 (0.04)	
Gonorrhoea						
Gonorrhoea at mpox diagnosis, n (%)[1]	220 (10.4)	147 (11.5)	73 (8.7)	0.037	NA	
Gonorrhoea in the 2 years before mpox, n (%)	650 (30.7)	403 (31.5)	247 (29.3)	0.29	1147 (5.1)	<0.0001
Number of Neisseria gonorrhoeae events in the 2 years before mpox, n (%)						
1	451 (21.3)	276 (21.6)	175 (20.8)		947 (4.2)	
2	160 (7.5)	103 (8.1)	57 (6.8)		158 (0.7)	
>2	39 (1.8)	24 (1.9)	15 (1.8)		42 (0.2)	
Chlamydia trachomatis						
Chlamydia at mpox diagnosis, n (%)[1]	117 (5.4)	69 (5.3)	49 (5.8)	0.62	NA	
Chlamydia in the 2 years before mpox, n (%)	426 (20.1)	269 (21.0)	157 (18.7)	0.18	802 (3.6)	<0.0001
Number of Chlamydia events in the 2 years before mpox, n (%)						
1	339 (16.0)	210 (16.4)	129 (15.3)		706 (3.1)	
2	75 (3.5)	54 (4.2)	21 (2.5)		93 (0.4)	
>2	12 (0.6)	5 (0.4)	7 (0.8)		3 (0.01)	
Lymphogranuloma venereum (LGV)						
LGV diagnosed at mpox diagnosis, n (%)[1]	25 (1.2)	10 (0.8)	15 (1.8)	0.037	NA	
LGV in the 2 years before mpox, n (%)	117 (5.5)	52 (4.1)	65 (7.7)	<0.0001	177 (0.8)	<0.0001
Number of LGV events in the 2 years before mpox, n (%)						
1	109 (5.1)	47 (3.7)	62 (7.4)		168 (0.8)	
2	7 (0.3)	5 (0.4)	2 (0.2)		8 (0.04)	
>2	1 (0.05)	0	1 (0.1)		1 (0)	

[1] Time window of +/- 8 weeks.

[2] Sexually transmitted infections in the time period from 01.05.2022 to 01.05.2022
Abbreviations: LGV, lymphogranuloma venereum; PWH, people with HIV; PWoH, people without HIV; STI, sexually transmitted infections.

Table S4. Clinical characteristics in people with HIV (PWH) in active follow-up in the PISCIS HIV cohort, according to available HIV-RNA.

	PWH with VL <50 copies/ml at mpox diagnosis N=561 (83.4)	VL ≥50 copies/ml at mpox diagnosis N=47 (7.0)	p-value
Demographics			
Male, n (%)	559 (92.2)	47 (100)	0.68
Age (years), median (IQR)	41 (35-46)	40 (34-47)	0.77
Birth area, n (%)			0.38
Spain	211 (37.6)	20 (42.6)	
Eastern Europe	12 (2.1)	2 (4.3)	
Western Europe and Northern America	78 (13.9)	2 (4.3)	
Africa	9 (1.6)	2 (4.3)	
Latin America	243 (43.3)	20 (42.6)	
Other	8 (1.4)	1 (2.1)	
Route of HIV transmission, n (%)			<0.001
MSM	528 (96.7)	38 (86.4)	
Heterosexual men	10 (1.8)	1 (2.3)	
IDU	4 (0.7)	5 (11.4)	
Unknown	4 (0.7)	0	
Mpox			
Asymptomatic, n (%)	14 (2.5)	3 (6.4)	0.12
Symptoms, n (%)			
Fever	306 (54.6)	25 (53.2)	0.86
Asthenia	159 (28.3)	11 (23.4)	0.47
Odynophagia	124 (22.1)	9 (19.2)	0.64
Muscular pain	97 (17.3)	8 (17.0)	0.96
Headache	100 (17.8)	9 (19.2)	0.82
Generalized lymphadenopathy	65 (11.6)	7 (14.9)	0.50
Localized lymphadenopathy	226 (40.3)	17 (36.2)	0.64
Anogenital exanthema	296 (52.8)	21 (44.7)	0.29
Oro-facial exanthema	175 (31.2)	12 (25.5)	0.42
Generalized exanthema	248 (44.2)	24 (51.1)	0.36
Type of exanthema			
Maculopapular	79 (14.1)	9 (19.2)	0.34
Vesicular	108 (19.3)	9 (19.2)	0.99
Pustular	112 (20.0)	15 (31.9)	0.053
Umbilical	54 (9.6)	5 (10.6)	0.82
Crusts	39 (7.0)	6 (12.8)	0.14
Haemorrhagic	3 (0.5)	0	0.62
Complications, n (%)			
Bacterial infections	5 (0.9)	0	0.52
Cornea infections	3 (0.5)	0	0.62
Pneumonia	0	0	
Proctitis	8 (1.4)	2 (4.3)	0.14
Hospitalization	8 (1.4)	4 (8.5)	0.001
HIV-related			
CD4 cell count at mpox, (cells/µL), median (IQR)	724 (543-907)	463 (326-913)	0.006
AIDS-defining event prior to mpox	41 (7.3)	9 (19.2)	0.005
Comorbidity			
Malignancy	32 (5.7)	3 (6.4)	0.85
Autoimmune disease	16 (2.9)	3 (6.4)	0.18
Inflammatory bowel disease	14 (2.5)	1 (2.1)	0.88
Multiple sclerosis	0	0	
History of psychiatric disease	169 (30.1)	20 (42.6)	0.08
Charlson comorbidity score at mpox,			0.002
0	445 (79.3)	36 (76.6)	
1	34 (6.1)	4 (8.5)	
2-3	75 (13.4)	3 (6.4)	
≥4	7 (1.3)	4 (8.5)	
